# M2 macrophage-induced lncRNA PCAT6 facilitates tumorigenesis and angiogenesis of triple-negative breast cancer through modulation of VEGFR2

**DOI:** 10.1038/s41419-020-02926-8

**Published:** 2020-09-09

**Authors:** Fang Dong, Shengnan Ruan, Jinlong Wang, Yun Xia, Kehao Le, Xiaoyun Xiao, Ting Hu, Qiong Wang

**Affiliations:** 1 Department of Breast and Thyroid Surgery, Union Hospital, Tongji Medical College, Huazhong University of Science and Technology, 430022 Wuhan, Hubei China; 2 Department of Orthopaedic, Union Hospital, Tongji Medical College, Huazhong University of Science and Technology, 430022 Wuhan, Hubei China; 3 Cancer Center, Union Hospital, Tongji Medical College, Huazhong University of Science and Technology, 430022 Wuhan, Hubei China

**Keywords:** Breast cancer, Cell biology

## Abstract

As a common female malignancy, triple-negative breast cancer (TNBC) is the most malignant subtype of breast cancers (BC). This study further studied the role of long noncoding RNA (lncRNA) prostate cancer-associated transcript 6 (PCAT6) in TNBC. Functional assays, including EdU, wound healing, transwell, and immunofluorescence staining, revealed the effect of PCAT6 on cell proliferation, migration, and EMT process. The tube-formation assay disclosed the function of PCAT6 on angiogenesis. In vivo assays were also established to explore the impact of PCAT6 on tumor growth and microangiogenesis. The results revealed that PCAT6 boosted TNBC cell proliferation, migration, and angiogenesis both in vitro and in vivo. Then, this study unveiled that M2 macrophage secreted VEGF to stimulate the upregulation of PCAT6, thus promoting angiogenesis in TNBC. Next, through bioinformatics analysis and mechanism assays, we identified that PCAT6 positively regulated VEGFR2 expression via ceRNA pattern and then participated in VEGFR/AKT/mTOR signaling pathway to accelerate angiogenesis. Moreover, PCAT6 bound USP14, a deubiquitinase, to induce the deubiquitination of VEGFR2. On the whole, M2 macrophage-induced upregulation of PCAT6 facilitates TNBC tumorigenesis through modulation of VEGFR2 expression via ceRNA and deubiquitination patterns.

## Introduction

Breast cancer (BC) is a leading cause of cancer mortality for females over the world. Triple-negative breast cancer (TNBC) takes up 15–25% of BC cases. It is characterized by a shortage of ER, PR, and HER2 receptors. Currently, radiotherapy and cytotoxic chemotherapy are the preferred therapeutic methods for TNBC patients at early or advanced stages^1^. However, the overall survival of TNBC patients remains unfavorable due to early relapse, aggressive tumor growth, and distant recurrence.

Long noncoding RNAs (lncRNAs) are longer than 200 nucleotides and are incapable to code proteins. Various studies have reported that lncRNAs exerted great effects in the biological and pathological processes via multiple mechanisms. A common lncRNA-mediated mechanism is the competitive endogenous RNA (ceRNA) pattern. ceRNA is a typical post-transcriptional pattern that lncRNAs compete with message RNAs (mRNAs) to bind microRNAs (miRNAs), thus antagonizing the inhibition of miRNAs on mRNAs. The ceRNA pattern was widely observed in TNBC progression. Silenced CCAT1 inhibited TNBC cell proliferation, migration, and invasion through endogenously sponging miR-218 to elevate ZFX expression^2^. LINC01133 promotes phenotypic and growth characteristics of cancer stem cell-like cells via mediating the miR-199a/FOXP2 axis in TNBC cells^3^. Linc-ZNF469-3 contributes to TNBC lung metastasis via modulation on the miR-574-5p/ZEB1 axis^4^. NAMPT-AS served as a ceRNA to rescue the degradation of NAMPT from miR-548b-3p, thus promoting TNBC tumor development via activating the mTOR pathway^5^. ARNILA serves as a ceRNA for Sox4 via endogenously sponging miR-204 to facilitate TNBC invasion and metastasis^6^.

LncRNAs are also reported to bind with specific RNA-binding proteins (RBPs) and served as the scaffold to have an indirect modulation on target genes. MIR2052HG binds to EGR1 and promotes its recruitment to the promoter region of LMTK3 in ERα-positive BC^7^. MACC1-AS1 competitively interacted with PTBP1 protein to regulate cell growth^8^. LINC01355 interacted with FOXO3 protein to repress transcription of CCND1 and to block BC growth and progression^9^. Lnc10 binds to QKI-5 protein and prevents germ cell apoptosis via the p38 MAPK pathway^10^. NAMPT-AS recruits POU2F2 protein to transcriptionally upregulate NAMPT in TNBC^5^.

Prostate cancer-associated transcript 6 (PCAT6) is a common oncogene in non-small-cell lung cancer^11^. In our current study, we detected the expression and functional role of PCAT6 in TNBC.

During BC progression, cancer cells interact with surrounding tissues to create a microenvironment^12^. Tumor-associated macrophages (TAMs) are immune effector cells. Typically, TAMs are recruited to tumor tissues and secrete abundant chemokines, cytokines, growth factors, and inflammatory mediators^13^. A number of studies have uncovered that TAMs in the tumor microenvironment are actually M2-like polarized macrophage^14^. TAMs predominantly polarized toward M2-like macrophages and promoted malignant tumor progression^15^. Zhu et al. pointed out that immune cell recruitment is reduced, while M2-like macrophages are increased in metastatic BC tumors^16^. Brummer et al. have revealed that selective targeting of CCR2 impaired BC tumor growth and invasion as well as decreased M2 macrophages-induced angiogenesis^17^. Chen et al. revealed that activated M2 macrophages are strongly associated with survival for both Asian and Western patients with BC^18^.

In this study, we explored the function of PCAT6 and the mechanism by which M2 macrophage induced the upregulation of PCAT6. Besides, the underlying mechanism of PCAT6 to regulate target genes was also the research object of our current study.

## Materials and methods

### Tissue samples

The ethical approval of this study was acquired from the Ethics Committee of Union Hospital, and written informed consents were provided by all participants. A total of 86 pairs of TNBC and adjacent normal tissue samples from patients who did not receive radiotherapy and chemotherapy before surgery were collected for this study between January, 2014 and March, 2019. Tissue samples were all snap-frozen in liquid nitrogen and preserved at −80 °C.

### Cell lines and reagents

Human normal breast epithelial cell (MCF-10A) and human TNBC cell lines (MDA-MB-231, MDA-MB-468, MDA-MB-436, HCC-1937) were available from the ATCC Cell Bank (Manassas, VA). Cell samples were cultivated in DMEM (Invitrogen, Carlsbad, CA), adding FBS (10%) and penicillin–streptomycin mixture (1%) under 37 °C and 5% CO_2_. Besides, cycloheximide (CHX; 5 µM) and MG132 (10 μM) were both purchased from Sigma-Aldrich (St. Louis, MO). Crenolanib (1 μm) was bought from Selleckchem (Houston, TX).

### Quantitative real-time polymerase chain reaction (qRT-PCR)

The total RNA was isolated from cells by using the Trizol Kit in line with specification (Invitrogen) for reverse transcription using Takara RT reagent (Takara, Shiga, Japan). Quantitative analysis was implemented via Step-One Plus Real-Time PCR System (Applied Biosystems, Foster City, CA). All results were processed with 2^−ΔΔCT^ method after normalizing to GAPDH or U6.

### Transmission electron microscopy (TEM)

At first, exosome pellets were re-suspended by 0.2 M phosphate buffer (200 μL) and mixed by 2% paraformaldehyde. TEM was applied to characterize the morphology of isolated exosomes as instructed (Hitachi HT7700, Tokyo, Japan).

### Subcellular fractionation

Subcellular fraction assay was carried out for the separation of cytosolic and nuclear fractions using the PARIS Kit (Invitrogen) as required. For quantifying PCAT6 content, GAPDH and U6 acted as cytoplasmic or nuclear controls.

### FISH

The designed FISH probe for PCAT6 was obtained from Ribobio Company (Guangzhou, China). Cells of MDA-MB-468 and MDA-MB-436 were prepared for hybridization with PCAT6-FISH probe, then incubated in DAPI solution for counterstain, and observed under a fluorescence microscope (Nikon, Tokyo, Japan).

### Plasmid transfection

To stably silence PCAT6 and USP14, the designed shRNAs (GenePharma Shanghai, China) and NC-shRNAs were constructed and transfected into MDA-MB-468 and MDA-MB-436 cells for 48 h using Lipofectamine 2000 (Invitrogen). Besides, the pcDNA3.1/VEGFR2, pcDNA3.1-PCAT6, and NC-pcDNA3.1 vectors, as well as miR-4723-5p and NC mimic/inhibitor, were all bought from GenePharma for plasmid transfection.

### EdU assay

After transfection, MDA-MB-468 and MDA-MB-436 cells were plated in a 96-well plate with 5 × 10^4^ cells every well, then subjected to EdU assay as required by the provider (Ribobio). DAPI dye was applied for counterstaining the cell nucleus; a fluorescence microscope was finally used.

### Colony-formation assay

MDA-MB-468 and MDA-MB-436 cells were plated into a 96-well plate at 500 cells/well and incubated for 14 days, then fixed by 4% paraformaldehyde. After 0.1% crystal violet staining, colonies were counted.

### Transwell invasion assay

Matrigel (Clontech, Madison, WI) was used to coated the transwell inserts (Corning, Corning, NY) for invasion assay. The lower chamber was filled with conditioned culture medium, and cells suspended in serum-free medium were added into the upper chamber for 48 h. After that, cells invading to bottom were counted visually via crystal violet dye and light microscope.

### Wound-healing assay

For scratch wound healing, processed cells were cultivated for 24 h in serum-free medium, then wounded by pipette tips. The culture medium was refreshed, and the distance of wound healing was recorded 24 h later and imaged.

### Western blot

Cells were lysed in RIPA lysis buffer, then the collected cell protein was separated on 12% SDS-PAGE and shifted onto PVDF membranes. After sealing in 5% skimmed milk, the diluted primary antibodies at 1:2000 were available from Abcam (Cambridge, MA) and used all night. Following three washes in TBST, membranes were probed with the diluted secondary antibodies at 1:5000 (Abcam) for 2 h. ECL detection system was used for monitoring signals as instructed (Pierce, Rockford, IL).

### Immunofluorescence (IF)

After TNBC cells adhered to the culture slides, cells were first rinsed in PBS and then fixed for 10 min, followed by the blockade in 5% BSA for 10 min. After incubation with the primary antibody against E-cadherin or N-cadherin in PBS, cells were washed in PBS for culturing with the second antibody. DAPI dye was finally used for nuclear detection.

### Tube-formation assay

Matrigel was evenly distributed to every well in a 96-well plate for 30 min at 37 °C. TNBC cells at early passage were prepared after transfection in serum-free medium and added 100 μl of cell suspension into each well. Tube formation was assessed under the microscope.

### Luciferase reporter assay

For VEGFR2 promoter-luciferase assay, MDA-MB-468 and MDA-MB-436 cells were co-transfected for 48 h with pGL3 reporter vector containing VEGFR2 promoter and pcDNA3.1-PCAT6 or pcDNA3.1-NC. Besides, cells were co-transfected with miR-4723-5p-mimics or NC-mimics and pmirGLO reporter vectors, including PCAT6-wt/mut and VEGFR2-wt/mut. Based on the protocol (Promega, Madison, WI), Luciferase Reporter Assay System was used to assess luciferase activity.

### RNA pull-down assay

Using Pierce Magnetic RNA-Protein Pull-Down Kit, RNA pull-down assay was achieved as required by the supplier (Thermo Fisher Scientific, Waltham, MA). The cell protein extracts were prepared to incubate with biotinylated PCAT6 probes and NC probes in magnetic beads. After RNA enrichment, qRT-PCR was conducted.

### RNA immunoprecipitation (RIP)

Using Magna RIP™ RNA-Binding Protein Immunoprecipitation Kit, RIP assay was accomplished as instructed by the provider (Millipore, Bedford, MA). Cell lysates were used for immunoprecipitation with the human Ago2 antibody (Millipore) or USP14 antibody (Abcam) in magnetic beads. IgG antibody (Millipore) was used in the control group.

### Co-immunoprecipitation (Co-IP)

After lysing in IP lysis buffer, cell lysates were collected and cultured with USP14, VEGFR2, or IgG antibody all night at 4 °C in constant speed, then magnetic beads were added for 1 h. After washing with IP lysis buffer, the eluted protein samples were subjected to western blot.

### Animal assay

Both xenograft tumor and metastatic models were constructed using the male BALB/C nude mice (6-week; Union Hospital), with the ethical approval from the Animal Research Ethics Committee of Union Hospital. For xenograft tumor analysis, mice were injected subcutaneously with 1 × 10^6^ transfected TNBC cells for 28 days, and tumor volume was monitored every fourth day. Mice were killed by cervical decapitation; tumors were then dissected for weigh detection and further analysis. For metastasis assay, mice were killed after 8-week of tail vein injection, and metastatic nodules were observed via hematoxylin and eosin (H&E) staining.

### Chick chorioallantoic membrane (CAM) assay

The fertilized chicken eggs were maintained at 37 °C with 60% humidity. Then, 1 × 10^6^ TNBC cells were inoculated on the CAM of a 10-day-old chick, and an artificial air sac was made under aseptic condition. After resealing the window, eggs were returned to the incubator. Microangiogenesis was observed under a microscope after 72 h.

### Immunohistochemistry (IHC)

The collected tumor tissue samples isolated from xenograft tumor assay were fixed by 4% paraformaldehyde and prepared to dehydrate and embed in paraffin. After cutting, the consecutive sections at 4-μm thick were incubated with the ki-67 antibody or PCNA antibody (Abcam) for IHC assay.

### Statistical analysis

All assays were bio-repeated in triplicate, and experimental results were expressed as the means ± standard deviation (S.D.). Student’s *t* test or one-way ANOVA was applied to determine statistical probabilities, with *P* value below 0.05 as significant levels. SPSS v.19.0 software (IBM Corp., Armonk, NY) was used for statistical analysis, and gene linear correlation was analyzed by Pearson correlation analysis.

## Results

### PCAT6 is upregulated in TNBC tissues and cells

**Fig. 1:**
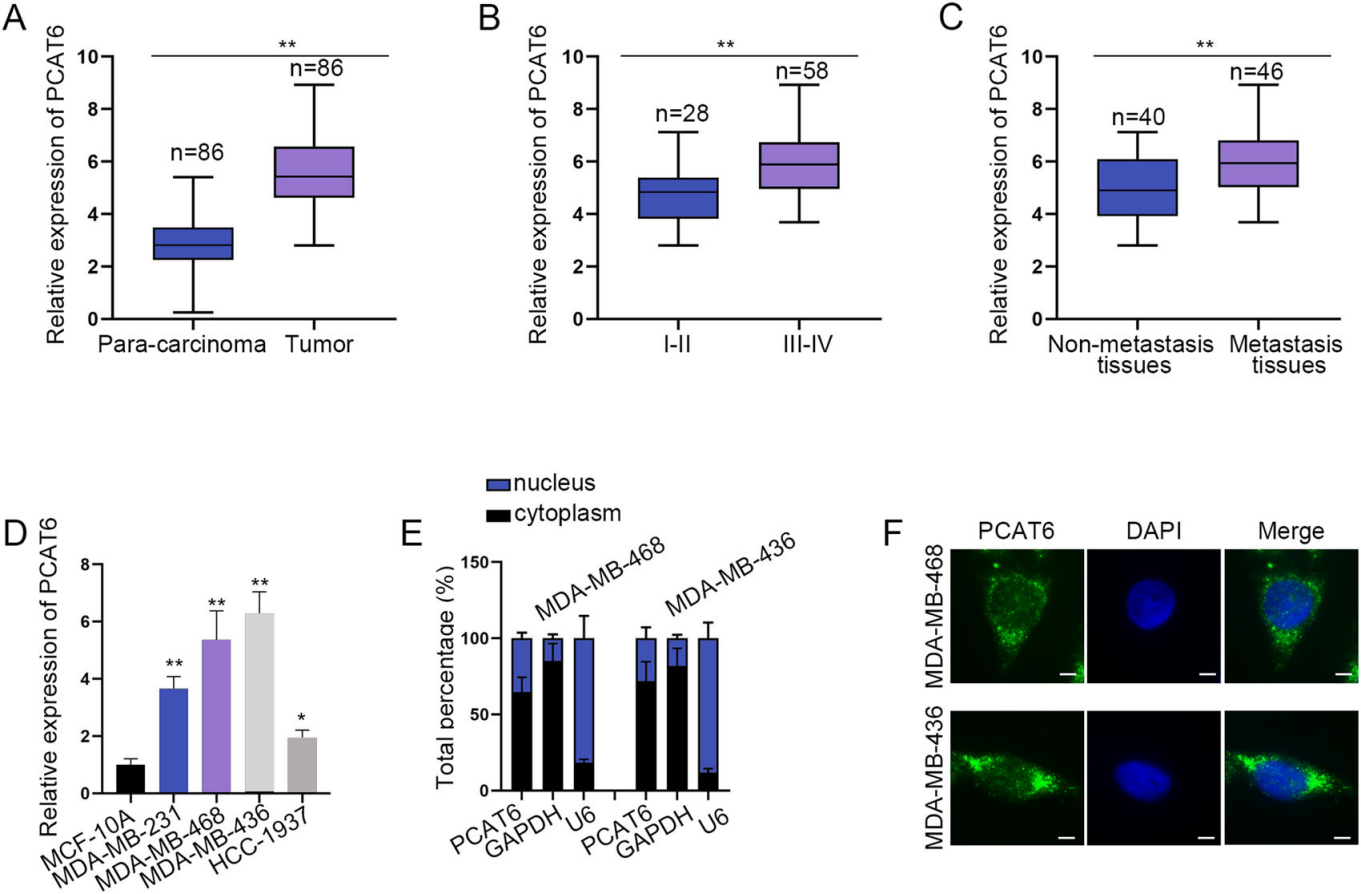
Upregulation of prostate cancer-associated transcript 6 (PCAT6) in triple-negative breast cancer (TNBC) tissues and cells. **a** qRT-PCR detected the relative expression of PCAT6 in TNBC tissues and para-carcinoma tissues. **b** qRT-PCR examined PCAT6 expression at different stages of TNBC tissues. **c** qRT-PCR evaluated PCAT6 expression in non-metastasis and metastasis tissues. Student’s *t* test. **d** qRT-PCR evaluated PCAT6 expression in TNBC cells and normal mammary epithelial cells. One-way ANOVA. **e**, **f** Subcellular fraction assay and FISH assay (scale bar: 10 μm) detected the subcellular location of PCAT6. *P < 0.05, **P < 0.01.

At first, the expression of PCAT6 in 86 pairs of TNBC tissues and paired nontumor tissues was examined. It was revealed that PCAT6 was significantly upregulated in tumor tissues than in para-carcinoma tissues (Fig. 1a). Then, PCAT6 expression in TNBC tissues obtained from patients at different stages was detected. The results revealed that PCAT6 expression was positively correlated with higher stages (Fig. 1b). Next, we figured out that metastatic tissues contained a higher expression of PCAT6 than non-metastatic tissues (Fig. 1c). After that, we examined the expression of PCAT6 in four TNBC cells and control cells. PCAT6 was noticeably upregulated in TNBC cells than in control cells (Fig. 1d). Since MDA-MB-468 and MDA-MB-436 presented the highest level of PCAT6, they were chosen for the following assays. Subsequently, we examined the cellular location of PCAT6. As demonstrated by a subcellular fraction and FISH assay, PCAT6 was distributed in both cytoplasm and nucleus of TNBC cells (Fig. 1e, f). In brief, PCAT6 is upregulated in TNBC tissues and cells, and might exert functions at the transcriptional or post-transcriptional level.

### PCAT6 drives TNBC cell proliferation, migration, invasion, and EMT process

**Fig. 2:**
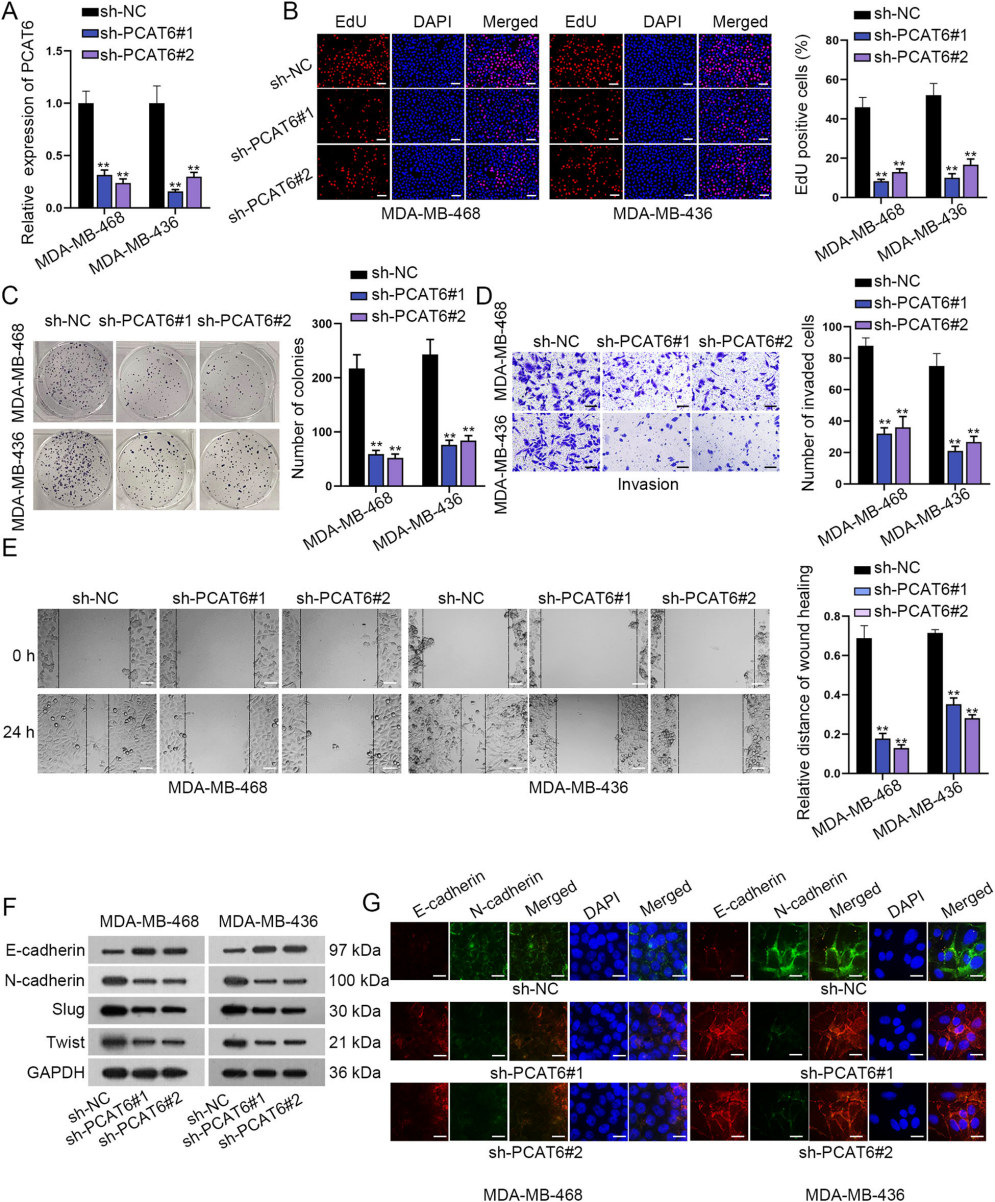
Prostate cancer-associated transcript 6 (PCAT6) boosted triple-negative breast cancer (TNBC) cell proliferation, invasion, and EMT. **a** qRT-PCR detected knockdown efficiency of PCAT6. **b**, **c** EdU (scale bar: 100 μm) and colony-formation assay evaluated the proliferation capacity of TNBC cells after knockdown of PCAT6. **d**, **e** Transwell (scale bar: 200 μm) and wound-healing assay (scale bar: 100 μm) examined the invasion capacity of TNBC cells after silencing PCAT6. **f** Western blot detected expression of E-cadherin, N-cadherin, Slug, and Twist by PCAT6 knockdown in TNBC cells. **g** Immunofluorescence staining assay was carried out to detect E-cadherin, N-cadherin positivity after silencing PCAT6 in TNBC cells (scale bar: 50 μm). **P < 0.01.

Loss-of-function assays were designed to identify the function of PCAT6 in TNBC cells. PCAT6 expression was firstly knocked down by shRNAs targeting PCAT6. The knockdown efficacy was verified in the qRT-PCR assay (Fig. 2a). Then, cell proliferation assays revealed that depletion of PCAT6 attenuated cell proliferation (Fig. 2b, c). Transwell invasion assay disclosed that cell invasion was remarkably reduced by silenced PCAT6 (Fig. 2d). Similarly, the wound-healing assay showed that silenced PCAT6 hampered cell migration (Fig. 2e). Furthermore, western blot analysis indicated that silenced PCAT6 caused a significant increase in E-cadherin level and a remarkable decrease in the levels of N-cadherin, Slug, and Twist (Fig. 2f and Supplementary File 1A). Finally, immunofluorescence (IF) staining assay was implemented to explore the positivity of E-cadherin and N-cadherin. The positivity of E-cadherin was elevated, while that of N-cadherin was reduced in PCAT6 silenced TNBC cells (Fig. 2g). According to these data, PCAT6 is an oncogene in TNBC via facilitating cell proliferation, migration, invasion, and EMT process.

### M2 macrophage secretes VEGF to upregulate PCAT6

**Fig. 3:**
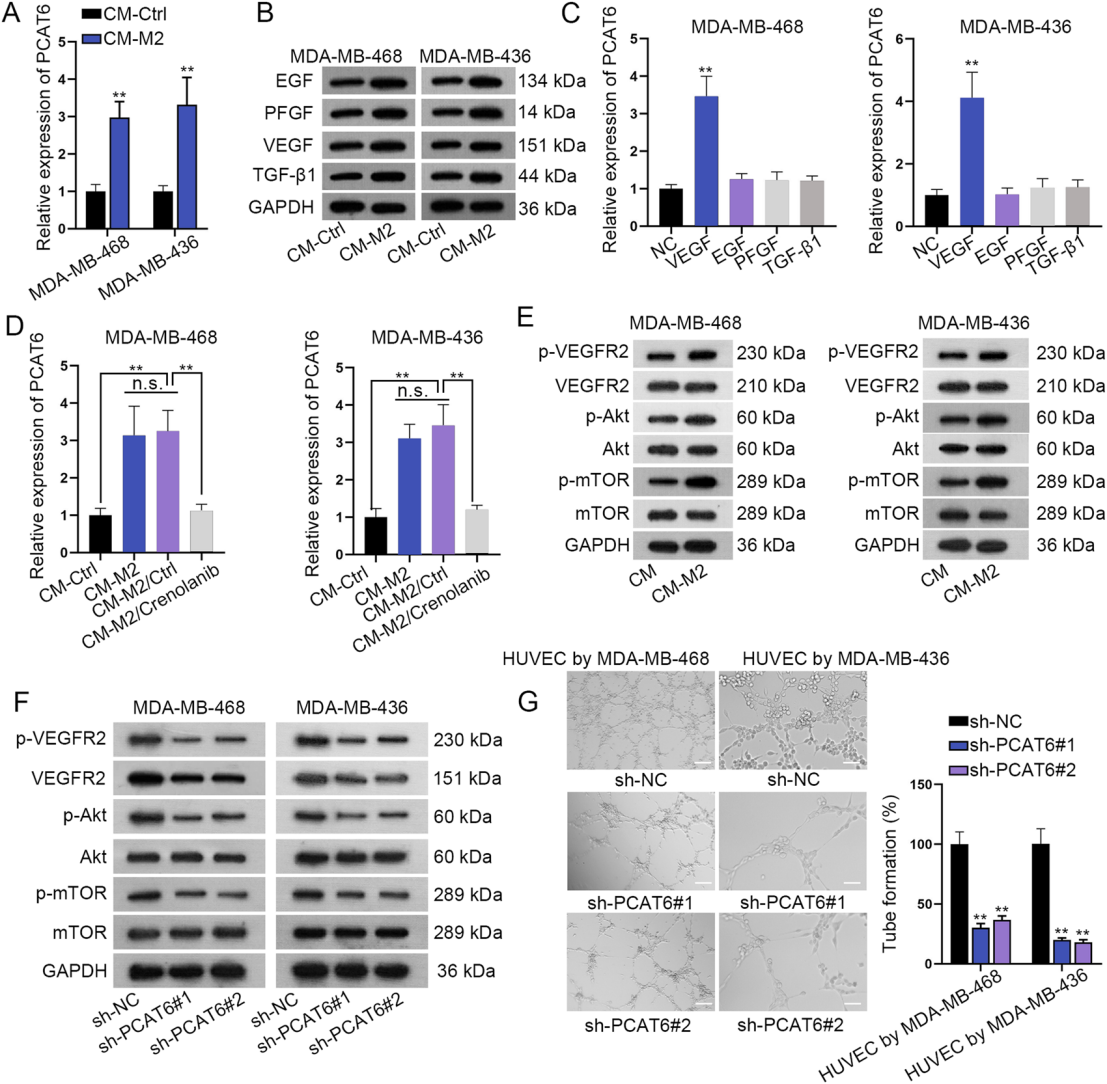
VEGF stimulated the upregulation of prostate cancer-associated transcript 6 (PCAT6) to promote angiogenesis in triple-negative breast cancer (TNBC) cells. **a** qRT-PCR detected the relative expression of PCAT6 in TNBC cells in CM-Ctrl and CM-M2 group. **b** Western blot detected the expression of EGF, PFGF, VEGF, and TGF-β1 in CM-Ctrl and CM-M2 group. **c** qRT-PCR detected PCAT6 expression in TNBC cells by treatment of EGF, PFGF, VEGF, and TGF-β1. **d** qRT-PCR detected PCAT6 expression in the CM-Ctrl, CM-M2, CM-M2-Ctrl, and CM-M2/Crenolanib group. **e** Western blot detected the expression of the VEGFR2/Akt/mTOR axis in CM-Ctrl and CM-M2 group. **f** Western blot evaluated the expression of the VEGFR2/Akt/mTOR axis by PCAT6 depletion in TNBC cells. **g** Tube-formation assay detected angiogenesis ability by PCAT6 knockdown in HUVEC cells (scale bar: 200 μm). **P < 0.01.

A previous study has reported that M2 macrophage promoted BC cell proliferation and migration in tumor microenvironment^15^. Here we wondered if M2 macrophage affected the expression of PCAT6 in TNBC cells. It was revealed that PCAT6 was significantly upregulated in cells cultured with an M2-conditioned medium than in control cells (Fig. 3a). Next, we sought to explore how M2 promoted PCAT6 expression. M2 macrophage was reported to secret growth factors, including EGF, PFGF, VEGF, and TGF-β1^19^. We wondered if PCAT6 expression was stimulated by these growth factors in TNBC cells. It was revealed in western blot assay that the protein levels of EGF, PFGF, VEGF, and TGF-β1 were significantly upregulated in the CM-M2 group than that in the control group (Fig. 3b and Supplementary File 1B). Then, PCAT6 expression was significantly upregulated by VEGF, but not by EGF, PFGF, and TGF-β1 (Fig. 3c). Subsequently, crenolanib, an inhibitor of VEGF, was used in CM-M2. It was revealed that PCAT6 expression was significantly upregulated in CM-M2, while crenolanib caused a significant decrease of PCAT6 expression in CM-M2 (Fig. 3d). VEGF is a specific angiogenesis factor and could stimulate endothelial tube formation to generate new vessels^20^. A previous study revealed that VEGF primarily activates the VEGFR2-mediated Akt–mTOR pathway to induce angiogenic responses^21^. Thus, we examined protein expression of p-VEGFR2, p-Akt, and p-mTOR in CM-M2 and control groups. It was revealed that p-VEGFR2, p-Akt, and p-mTOR expression was significantly upregulated in CM-M2 than in the control group. In the meanwhile, total VEGFR2, Akt, and mTOR were not impacted (Fig. 3e and Supplementary File 1C). When PCAT6 was knocked down, p-VEGFR2, total VEGFR2, p-Akt, and p-mTOR expression was significantly reduced in TNBC cells (Fig. 3f and Supplementary File 1D). Next, the tube-formation assay was conducted. Depletion of PCAT6 remarkably reduced tube-formation efficiency in HUVECs (Fig. 3g). Exosomes derived from M2 macrophages were detected by TEM (Supplementary Fig. S1A). Moreover, TNBC cells treated with M2 exosome presented a higher PCAT6 level than control cells (Supplementary Fig. S1B). Thus, we came to the conclusion that M2 macrophage secretes VEGF to upregulate PCAT6, and further induced the VEGFR2/Akt/mTOR pathway and angiogenesis.

### PCAT6 acts as a sponge for miR-4723-5p to upregulate VEGFR2

**Fig. 4:**
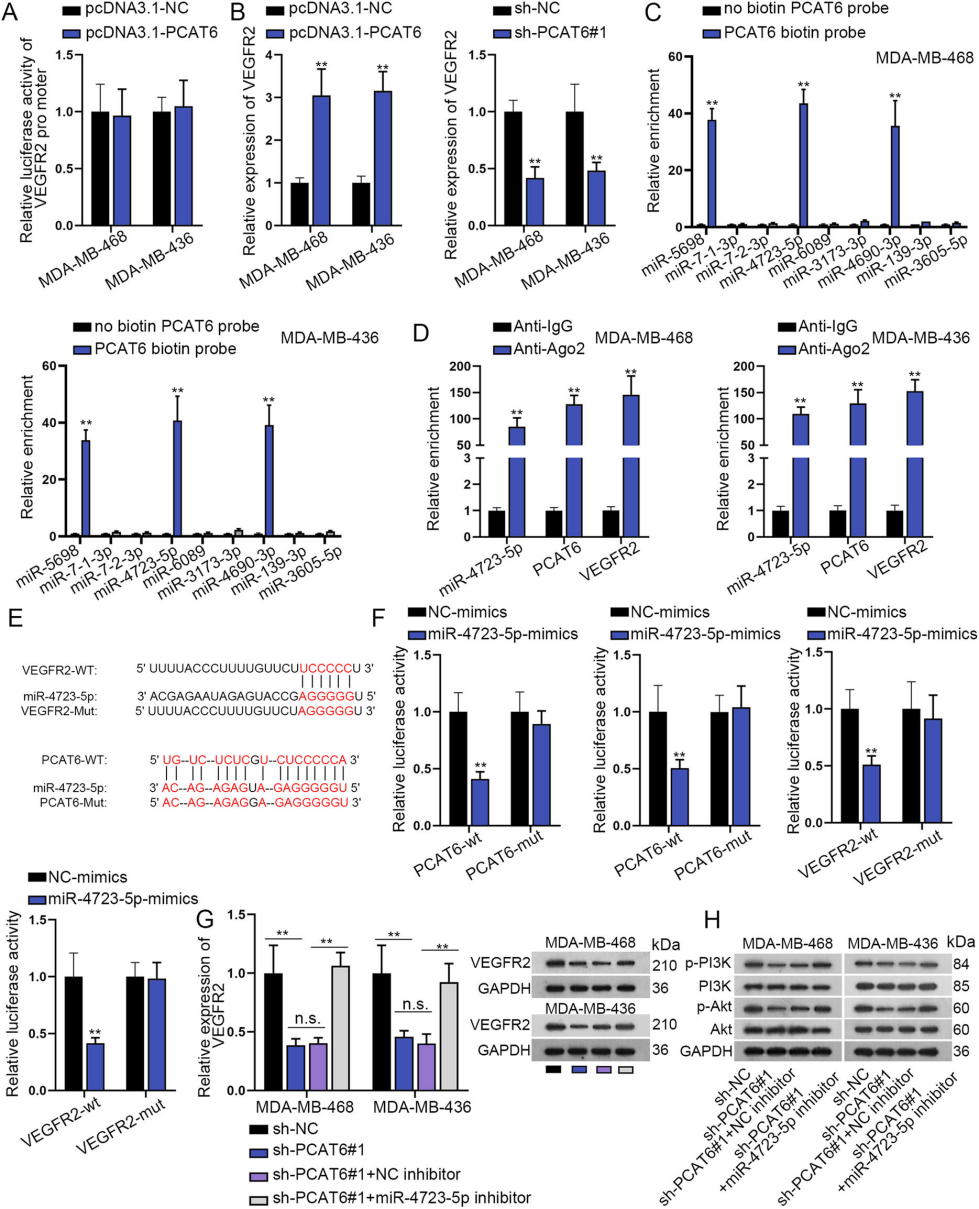
Prostate cancer-associated transcript 6 (PCAT6) post-transcriptionally regulates VEGFR2 expression. **a** Luciferase reporter assay was performed to examine the luciferase activity of the VEGFR2 promoter by knockdown of PCAT6. **b** qRT-PCR detected the relative expression of VEGFR2 by PCAT6 depletion or upregulation in triple-negative breast cancer (TNBC) cells. **c** RNA pull-down assay detected relative enrichment of putative microRNAs (miRNA)s pulled down by biotin-labeled PCAT6. **d** RIP assay measured the relative expression of miR-4723-5p, PCAT6, and VEGFR2 pulled down by anti-IgG and anti-Ago2. **e** Binding sequences of PCAT6/VEGFR2 (wild and mutant) and miR-4723-5p. **f** Luciferase reporter assay was performed to examine the luciferase activity of wild/mutant PCAT6 and VEGFR2 by miR-4723-5p overexpression. **g** qRT-PCR and western blot examined VEGFR2 expression in different groups. **h** Western blot analysis of the Akt/mTOR axis-related proteins. **P < 0.01. n.s. no significance.

Since we have previously verified that PCAT6 regulated the total protein expression of VEGFR2, we sought to examine how PCAT6 regulated VEGFR2. The first luciferase reporter assay revealed that PCAT6 had no impacts on the transcription of VEGFR2 (Fig. 4a). Results from qRT-PCR assay revealed that the upregulation of PCAT6 positively regulated the mRNA level of VEGFR2 (Supplementary Fig. S2A and Fig. 4b). We supposed that whether cytoplasmic PCAT6 modulated VEGFR2 via the ceRNA pattern. Based on miRmap and lncBase, nine miRNAs were revealed to bind both PCAT6 and VEGFR2 (Supplementary Fig. S2B). Among these nine miRNAs, miR-5689, miR-4723-5p, and miR-4690-3p were significantly pulled down by biotin-labeled PCAT6, while no productions were observed in non-biotin-labeled PCAT6 (Fig. 4c). Further, expression levels of miR-5689, miR-4723-5p, and miR-4690-3p in TNBC tissues and adjacent nontumor tissues were detected. Results revealed that only miR-4723-5p was downregulated in TNBC tissues (Supplementary Fig. S2C). Pearson correlation test revealed a positive correlation between PCAT6 and VEGFR2, as well as a negative correlation between VEGFR2 and miR-4723-5p (Supplementary Fig. S2D). RIP assay further revealed that PCAT6, miR-4723-5p, and VEGFR2 were abundantly enriched in the anti-Ago2 group, while no production was significantly observed in the anti-IgG group (Fig. 4d). Then, we enhanced the expression of miR-4723-5p, and the overexpression efficiency of miR-4723-5p was verified in the qRT-PCR assay (Supplementary Fig. S2E). MiR-4723-5p expression was noticeably downregulated in TNBC cells than in control cells (Supplementary Fig. S2F). MiR-4723-5p and PCAT6 could not impact mRNA expression of each other (Supplementary Fig. S2G). Subsequently, the binding sites of miR-4723-5p on PCAT6 were predicted from lncBase, and that of miR-4723-5p and VEGFR2 were obtained from miRmap (Fig. 4e). We mutated the binding sites for the next luciferase reporter assay. Then, it was uncovered that the luciferase activity of wild PCAT6 and VEGFR2 vectors were reduced by miR-4723-5p overexpression. In the meanwhile, the mutation caused the abrogation on changes of luciferase activity of PCAT6 and VEGFR2 (Fig. 4f). Moreover, qRT-PCR assay revealed that VEGFR2 mRNA expression was reduced by silenced PCAT6, while downregulation of miR-4723-5p completely rescued such effects. Intriguingly, western blot analysis revealed that downregulation of miR-4723-5p partially rescued the effects of PCAT6 on VEGFR2 protein expression (Fig. 4g and Supplementary File 2A). This phenomenon indicated that PCAT6 regulated VEGFR2 protein in another way. Importantly, miR-4723-5p partially rescued the effects of PCAT6 on the PI3K/Akt pathway (Fig. 4h and Supplementary File 2B). To sum up, PCAT6 serves as the ceRNA of VEGFR2 via sponging miR-4723-5p.

### PCAT6 recruits USP15 to stabilize VEGFR2

**Fig. 5:**
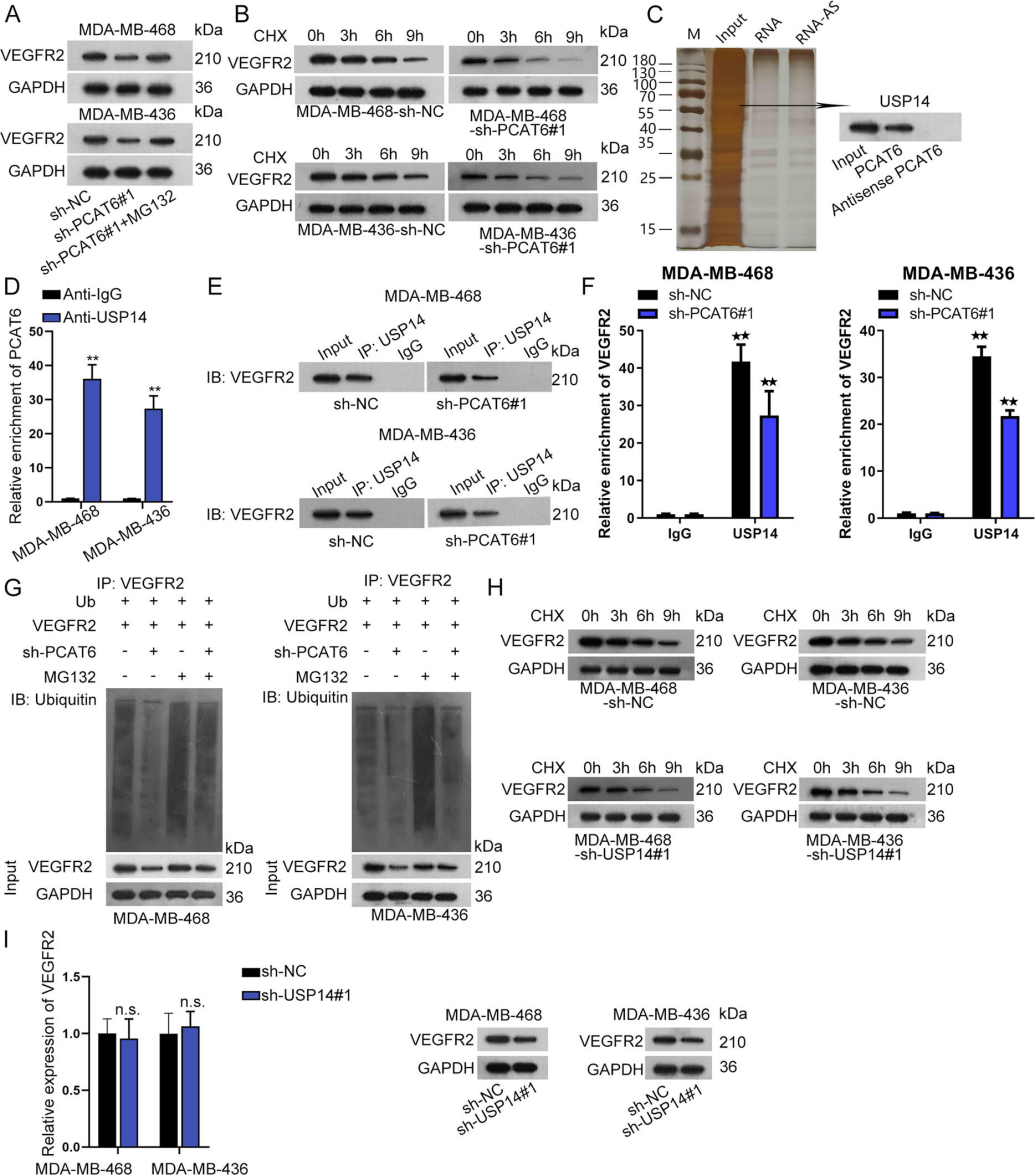
Prostate cancer-associated transcript 6 (PCAT6) recruited USP14 to stabilize VEGFR2. **a** Western blot examined VEGFR2 expression in the sh-NC, sh-PCAT6#1, and sh-PCAT6#1 + MG132 group. **b** Western blot evaluated VEGFR2 expression in the sh-NC and sh-PCAT6#1 group by treatment of cycloheximide (CHX). **c** RNA pull-down silver staining and western blot revealed USP14 as the protein-binding PCAT6. **d** RIP assay revealed enrichment of PCAT6 pulled down by anti-IgG and anti-USP14. **e** Co-IP revealed expression of VEGFR2 pulled down by USP14 using USP14 as IP and VEGFR2 as IB. **f** RIP assay revealed enrichment of VEGFR2 pulled down by USP14 when PCAT6 was knocked down. **g** Ubiquitination assay of VEGFR2 when PCAT6 was silenced. **h** Western blotting analysis of VEGFR2 expression in sh-NC and sh-USP14#1 group by treatment of CHX. **i** qRT-PCR and western blot examined VEGFR2 expression by USP14 knockdown. **P < 0.01. n.s. no significance.

In this section, we sought to examine how PCAT6 regulated VEGFR2 protein besides from ceRNA pattern. MG132 is a common inhibitor for proteasome^22^. We figured out that, by treatment of MG132, silenced PCAT6 could not impact the protein level of VEGFR2 (Fig. 5a and Supplementary File 2C). Then, we used cyclohexane (CHX) to treat cells. As was revealed in Fig. 5b and Supplementary File 2D, when PCAT6 was knocked down, VEGFR2 degraded at a faster speed than in the control group. We conducted the RNA pull-down assay and carried out mass spectrometry as well as western blot assay. The result revealed USP14, a deubiquitinating enzyme, as the PCAT6-binding protein (Fig. 5c). Also, PCAT6 is predicted as the RBP for PCAT6 based on the ENCORI database. The following RIP assay revealed that PCAT6 was significantly enriched in the anti-USP14 group (Fig. 5d). Further, a Co-IP assay was conducted using USP14 as the IP and VEGFR2 as the IB. It was disclosed that when PCAT6 was knocked down, VEGFR2 pulled down by USP14 was reduced compared to the control group (Fig. 5e and Supplementary File 2E). Moreover, when PCAT6 was silenced, the relative enrichment of VEGFR2 in USP14 was significantly reduced (Fig. 5f). After that, ubiquitination experiments were conducted. It was revealed that silenced PCAT6 caused more ubiquitination of VEGFR2 (Fig. 5g). Also, VEGFR2 degraded at a faster speed by CHX treatment when USP14 was knocked down (Fig. 5h and Supplementary File 2F). Finally, we verified the depletion efficiency of USP14 and figured out that USP14 did not impact the expression of PCAT6 (Supplementary Fig. S2H, I). Also, silenced USP14 had no influence on VEGFR2 mRNA but caused a remarkable decrease in protein level of VEGFR2 (Fig. 5i and Supplementary File 2G). According to all these findings, we concluded that PCAT6 recruits USP15 to impede the ubiquitination of VEGFR2.

### PCAT6 facilitates TNBC cell proliferation, migration, invasion, EMT, and angiogenesis through the VEGFR2/Akt/mTOR axis

**Fig. 6:**
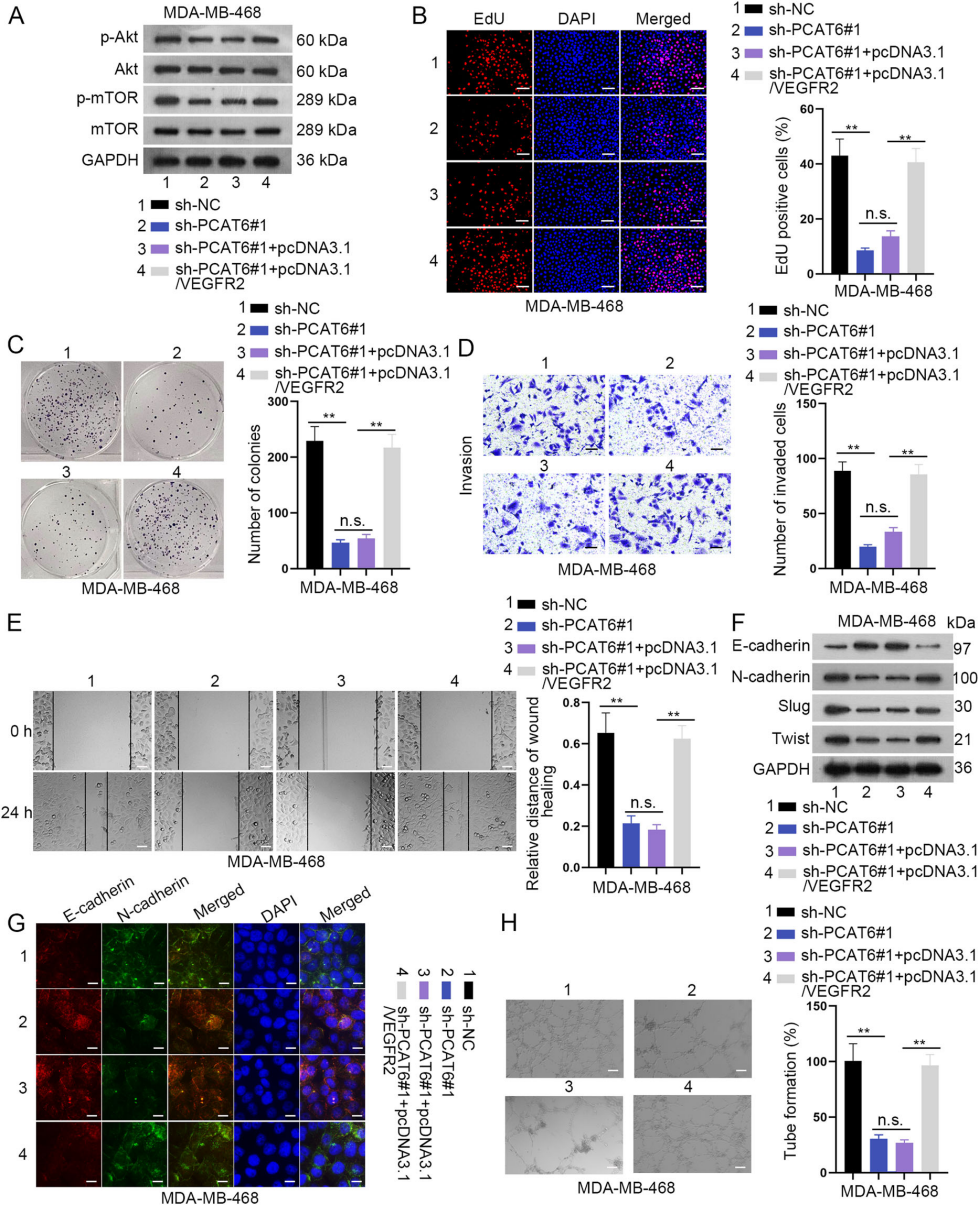
VEGFR2 was required in prostate cancer-associated transcript 6 (PCAT6)-mediated triple-negative breast cancer (TNBC) cell proliferation, invasion, EMT, and angiogenesis. **a** Western blotting of the Akt/mTOR axis in four groups: sh-NC, sh-PCAT6#1, sh-PCAT6#1 + pcDNA3.1, and sh-PCAT6#1 + pcDNA3.1/VEGFR2 group. **b**, **c** EdU (scale bar: 100 μm) and colony-formation assay revealed TNBC cell proliferation ability in four groups. **d**, **e** Transwell (scale bar: 200 μm) and wound-healing assay (scale bar: 100 μm) demonstrated TNBC cell invasion ability in four groups. **f**, **g** Western blot and immunofluorescence staining assay revealed TNBC cell EMT ability in four groups. **h** Tube-formation assay illustrated TNBC angiogenesis ability in four groups (scale bar: 200 μm). **P < 0.01. n.s. no significance.

Rescue assays were subsequently implemented to verify the function of the PCAT6/VEGFR2 axis. The first western blot assay revealed that VEGFR2 completely rescued the suppressive effects of silenced PCAT6 on the Akt/mTOR axis (Fig. 6a and Supplementary File 3A). EdU and colony-formation assays revealed that VEGFR2 completely restored the inhibitory effects of silenced PCAT6 on cell proliferation ability (Fig. 6b, c). Also, the repressive effects of PCAT6 on cell invasion and migration were completely counteracted by VEGFR2 (Fig. 6d, e). Subsequently, western blot and IF staining revealed that the EMT process was impaired by downregulated PCAT6, but that was completely rescued by upregulation of VEGFR2 (Fig. 6f, g and Supplementary File 3B). Finally, the tube-formation assay revealed that the suppressive influence of downregulated PCAT6 on tube-formation efficiency was completely restored by VEGFR2 (Fig. 6h). On the whole, VEGFR2 completely rescued the effects of PCAT6 on TNBC cell proliferation, migration, invasion, EMT, and angiogenesis via the Akt/mTOR axis.

### PCAT6 promotes tumor growth in vivo

**Fig. 7:**
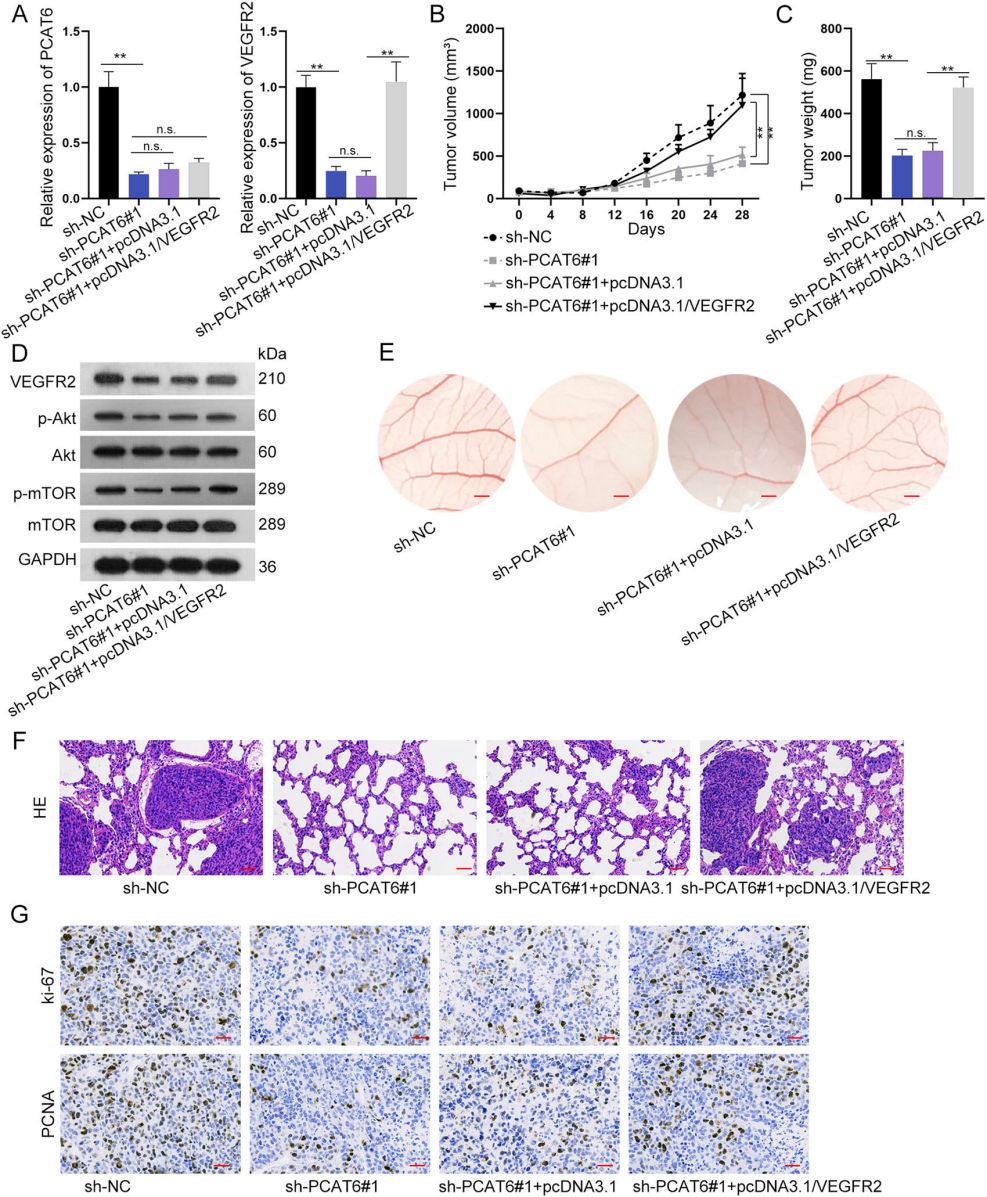
Prostate cancer-associated transcript 6 (PCAT6) and VEGFR2 facilitated triple-negative breast cancer (TNBC) tumor growth in vivo. **a** qRT-PCR detected PCAT6 and VEGFR2 expression in xenograft tumors. **b**, **c** Mice xenograft tumors: changes in tumor volume and weight were revealed in different groups. **d** Western blotting analysis of the VEGFR2/Akt/mTOR axis. **e** Microangiogenesis of chick chorioallantoic membrane (CAM) (scale bar: 200 μm). **f** HE staining assay revealed the morphology of non-metastasis tumor and metastasis tumor (scale bar: 100 μm). **g** Immunocytochemistry detected Ki-67 and PCNA positivity (scale bar: 100 μm). *P < 0.01. n.s. no significance.

In this section, the in vivo assays were conducted to verify the function of PCAT6 on tumor growth. As was revealed in the qRT-PCR assay, PCAT6 expression was significantly reduced by sh-PCAT6#1, and upregulation of VEGFR2 could not rescue the expression of PCAT6. VEGFR2 expression was significantly reduced by the downregulation of PCAT6, and was enhanced by the co-transfection of pcDNA3.1/VEGFR2 (Fig. 7a). The xenograft tumors in sh-NC, sh-PCAT6#1, sh-PCAT6#1 + pcDNA3.1, and sh-PCAT6#1 + pcDNA3.1/VEGFR2 were exhibited (Fig. 7b). Figure 7c, d and Supplementary File 3C demonstrated that downregulated PCAT6 reduced tumor volume and weight, while upregulated VEGFR2 exerted the opposite effects. Western blot assay revealed that expression of the VEGFR2/Akt/mTOR axis was hampered by the depletion of PCAT6. However, such effects were completely rescued by overexpression of VEGFR2. To further explore the anti-angiogenic activity of PCAT6, in vivo CAM neovascularization model was established. The microvessels of CAM decreased by silencing PCAT6, while pcDNA3.1/VEGFR2 enhanced microvessels of CAM (Fig. 7e). Further, HE staining of lung metastasis node morphology revealed that silenced PCAT6 inhibited metastasis while upregulated VEGFR2 promoted metastasis (Fig. 7f). IHC assay revealed that Ki-67 and PCNA positivity was significantly reduced by silenced PCAT6 and was enhanced by upregulated VEGFR2 (Fig. 7g).

According to all these data, M2 macrophage secretes VEGF to induce PCAT6 upregulation. PCAT6 promoted TNBC cell proliferation, migration, invasion, EMT, and angiogenesis, as well as tumor growth and metastasis via upregulation of VEGFR2 through sponging miR-4723-5p and recruiting USP14.

## Discussion

Our previous research revealed that M2 macrophage containing exosome transmitted PCAT6 promoted BC cell proliferation and migration. Based on that, this study went on to explore the function and mechanism of PCAT6 in TNBC. It was revealed that PCAT6 promoted TNBC cell proliferation, migration, invasion, and EMT. Since M2 macrophage could secret growth factors including EGF, PFGF, VEGF, and TGF-β1^19^, we then identified that PCAT6 expression was upregulated by VEGF secreted from M2 macrophage. VEGF is reported to activate the VEGFR2-mediated Akt/mTOR pathway to induce angiogenic responses^21^. Trinh et al. revealed that VEGF-A signaling serves as the stimulator of the AKT/mTOR pathway via VEGFR2 activation on epithelial ovarian cancer cells^23^. The Akt/mTOR pathway is a common oncogenic pathway in various cancers, BC included. HA-ADT inhibits PI3K/AKT/mTOR and Ras/Raf/MEK/ERK pathways to repress BC cell proliferation, viability, migration, and invasion^24^. GPNMB triggers the PI3K/AKT/mTOR pathway and β-catenin activity to augment BC initiation and growth^25^. Besides, VEGFR2 is commonly researched in BC. Di Mauro C et al. put forward that the Hedgehog pathway regulates VEGF/VEGFR2 loop on TNBC cell surface and orchestrates tumor vascularization in a paracrine manner to promote TNBC progression^26^. Zhang et al. disclosed that ACE2 hinders BC angiogenesis via inhibition on the VEGFa/VEGFR2/ERK pathway axis^27^. Then, it was uncovered in our study that PCAT6 induced the upregulation of VEGFR2 to trigger the VEGFR2/Akt/mTOR pathway and stimulate angiogenesis.

Subsequently, we sought to examine how PCAT6 regulated VEGFR2. PCAT6 was mainly distributed in the cytoplasm and nucleus of TNBC cells. PCAT6 did not transcriptionally influence VEGFR2 but positively regulated the mRNA level of VEGFR2. Here, we hypothesized whether PCAT6 regulated VEGFR2 via the ceRNA pattern. By means of bioinformatics analysis and a series of mechanism assays, PCAT6 served as the ceRNA of VEGFR2 via endogenously sponging miR-4723-5p. MiR-4723-5p has not been studied in any diseases before. This study only focused on the mechanism of miR-4723-5p and PCAT6/VEGFR2. However, the specific function of miR-4723-5p in TNBC remained unexplored, which is the disadvantage of this study. Importantly, we figured out that miR-4723-5p completely rescued the effects of PCAT6 on VEGFR2 at the mRNA level, but not at the protein level. This phenomenon indicated that PCAT6 regulated protein expression of VEGFR2 in another way.

It was subsequently revealed that the proteasome inhibitor suppressed the impacts of silenced PCAT6 on VEGFR2 expression. Further, VEGFR2 degraded at a faster speed when PCAT6 was silenced by CHX treatment. Since lncRNAs could recruit proteins with specific functions, here we wondered if PCAT6 bound to specific proteins and thus promoted stabilization of VEGFR2. Then, we found out that PCAT6 recruited USP14 to VEGFR2. Xia et al. have revealed that USP14 impairs the sensitivity of BC cells to enzalutamide^28^. MiR-124a targets USP14 to hinder stemness and promotes gefitinib sensitivity in non-small cell lung cancer cells^29^. Also, a previous study revealed that USP14 promotes cell proliferation via stabilizing AR in a deubiquitination manner in ER-negative BC cells^30^. PSMD2 regulated ubiquitin–proteasome degradation of p21 and p27 with the cooperation of USP14^31^. Ubiquitination is a common way for the degradation of proteins. This study uncovered that proteasomes recruit USP14 to inhibit the ubiquitination of VEGFR2. Similar to USP14, ATXN3 was identified as a novel deubiquitinating enzyme of KLF4 to promote BC metastasis^32^. USP1 promotes deubiquitination and stabilization of KPNA2 to facilitate lung metastasis of BC^33^. USP37 interacted with and stabilized Gli-1 to facilitate stemness, cell invasion, and EMT in BC via activation of the Hedgehog pathway^34^. Suppression of USP27X destabilizes Snail1 to impair BC cell EMT process and enhance cell sensitivity to chemotherapy^34^.

On the whole, it was first revealed in this study that M2 macrophage secreted VEGF to induce the upregulation of PCAT6. PCAT6 sponged miR-4723-5p and recruited USP14 to enhance the stabilization of VGEFR2 and induce the Akt/mTOR axis. PCAT6 promoted TNBC tumorigenesis, angiogenesis, tumor growth, and metastasis via the upregulation of VGEFR2, which might shed new insight into TNBC treatment.

## Supplementary information

Supplementary figure 1

Supplementary figure 2

Supplementary figures legends

Supplementary Table 1

Supplementary file 1

Supplementary file 2

Supplementary file 3

Supplementary files legends
