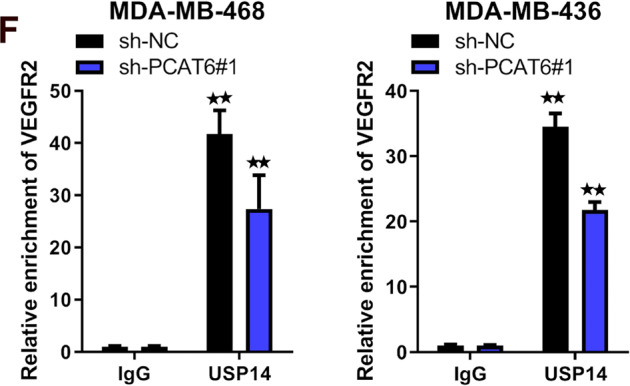# Correction: M2 macrophage-induced lncRNA PCAT6 facilitates tumorigenesis and angiogenesis of triple-negative breast cancer through modulation of VEGFR2

**DOI:** 10.1038/s41419-022-05203-y

**Published:** 2022-08-30

**Authors:** Fang Dong, Shengnan Ruan, Jinlong Wang, Yun Xia, Kehao Le, Xiaoyun Xiao, Ting Hu, Qiong Wang

**Affiliations:** 1 Department of Breast and Thyroid Surgery, Union Hospital, Tongji Medical College, Huazhong University of Science and Technology, 430022 Wuhan, Hubei China; 2 Department of Orthopaedic, Union Hospital, Tongji Medical College, Huazhong University of Science and Technology, 430022 Wuhan, Hubei China; 3 Cancer Center, Union Hospital, Tongji Medical College, Huazhong University of Science and Technology, 430022 Wuhan, Hubei China

**Keywords:** Breast cancer, Cell biology

Correction to: *Cell Death and Disease* 10.1038/s41419-020-02926-8, published online 09 September 2020

The original version of this article contained a mistake. The authors detected an error in Figure 5F of the published version of the article. The two pictures are duplicated, which were due to copy-paste errors in the final phase of preparing the figure for the submitted manuscript. The original article has been corrected.