# Supplementary figures and images for: M2 macrophage-induced lncRNA PCAT6 facilitates tumorigenesis and angiogenesis of triple-negative breast cancer through modulation of VEGFR2
Source: Cell Death Dis. 2020 Sep 9;11(9):728. doi: 10.1038/s41419-020-02926-8 (PMC7481779; doi:10.1038/s41419-020-02926-8)

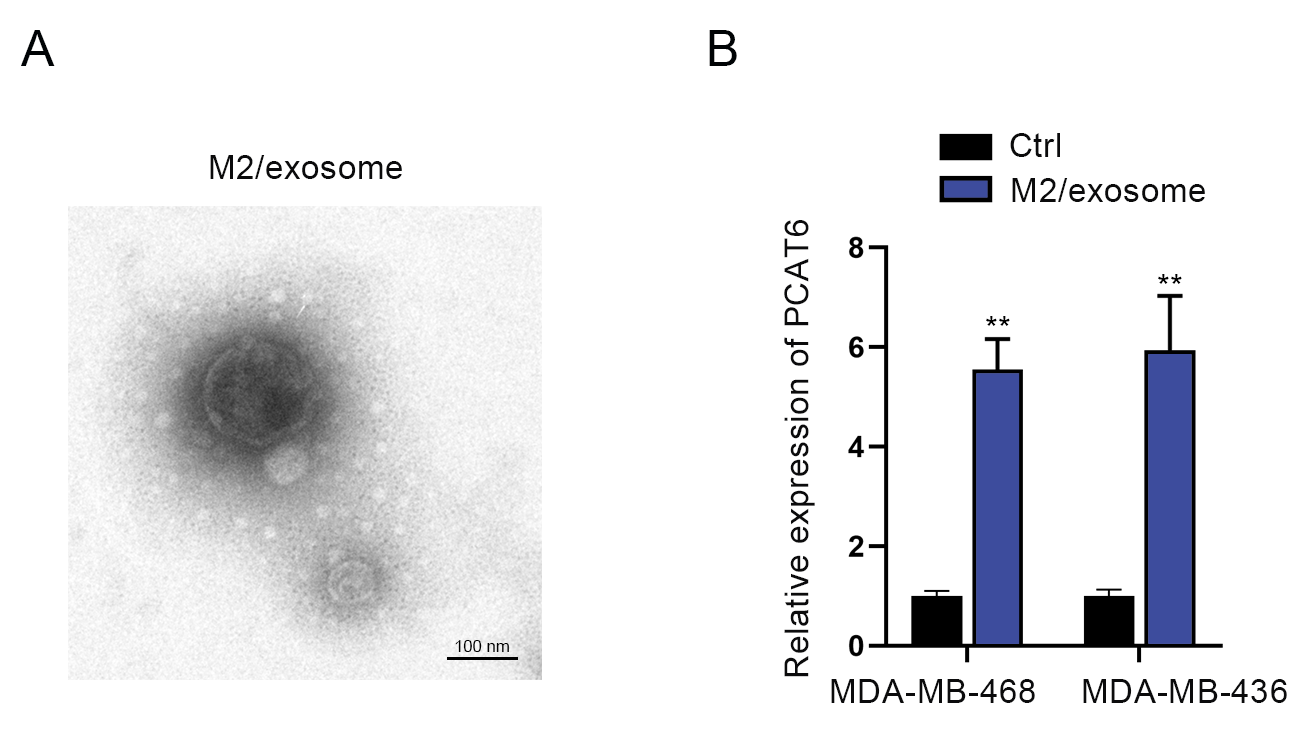

Supplement: Supplementary file 1 — Supplementary figure 1 [file 41419_2020_2926_MOESM1_ESM.tif]

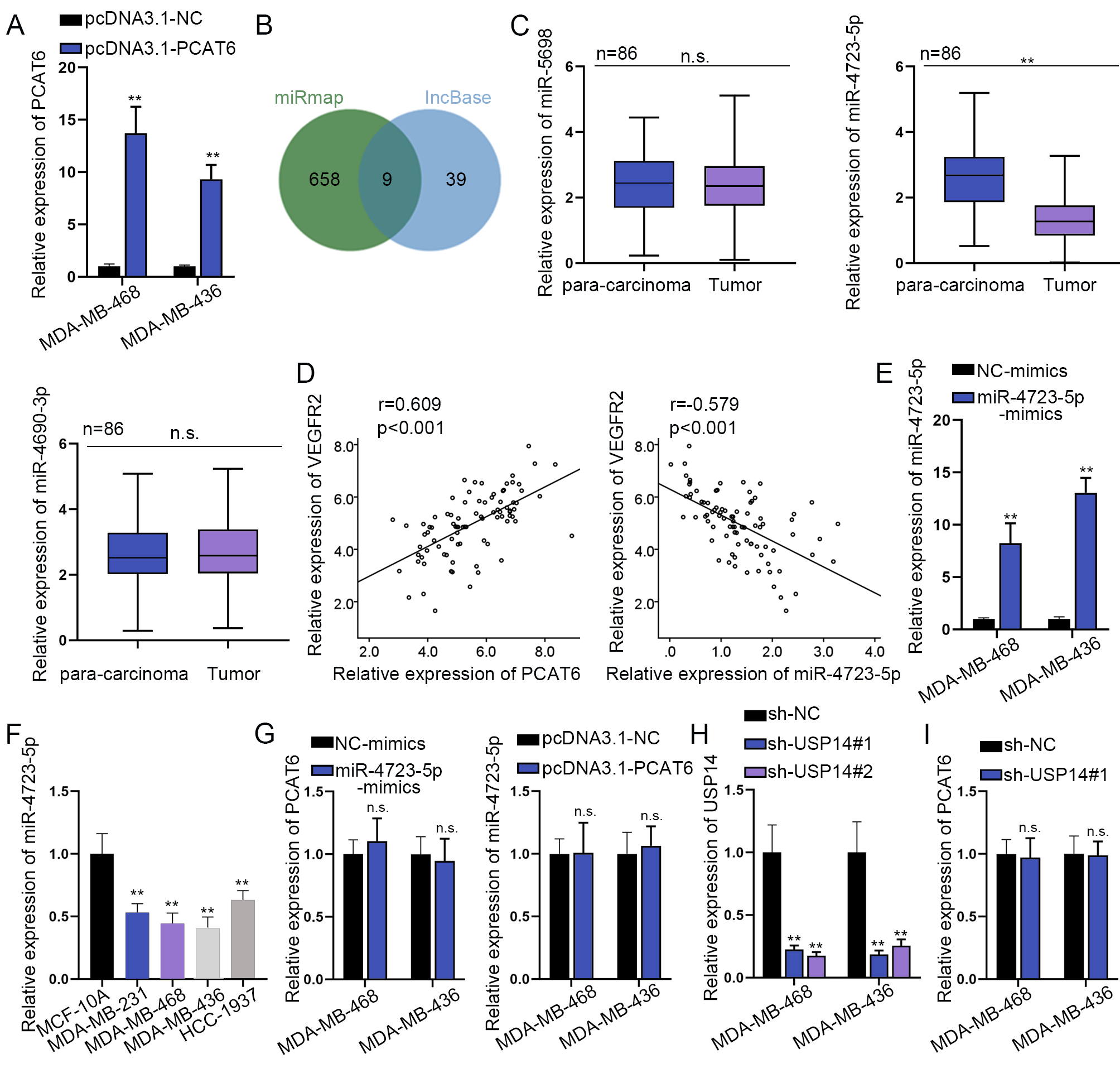

Supplement: Supplementary file 2 — Supplementary figure 2 [file 41419_2020_2926_MOESM2_ESM.tif]

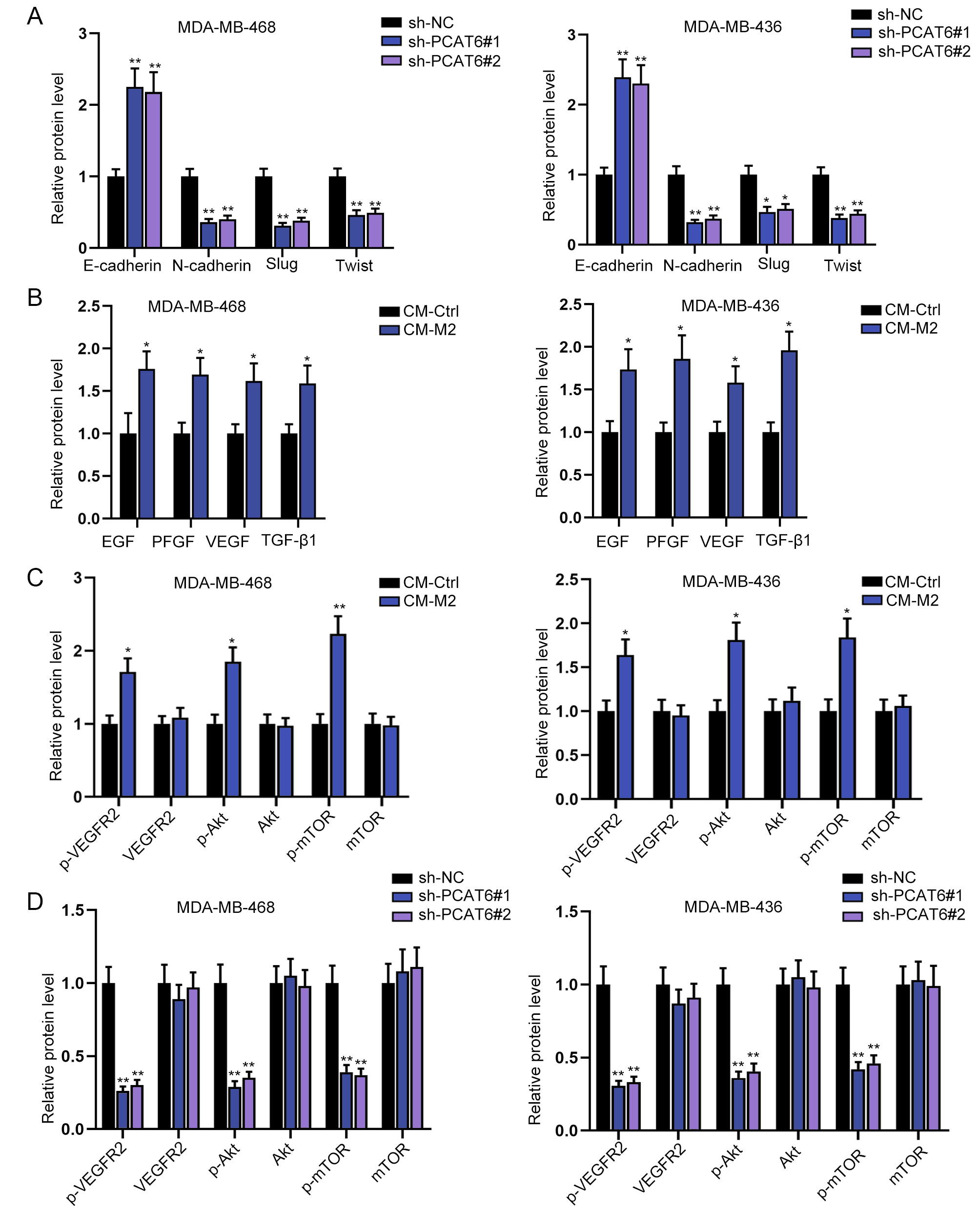

Supplement: Supplementary file 5 — Supplementary file 1 [file 41419_2020_2926_MOESM5_ESM.tif]

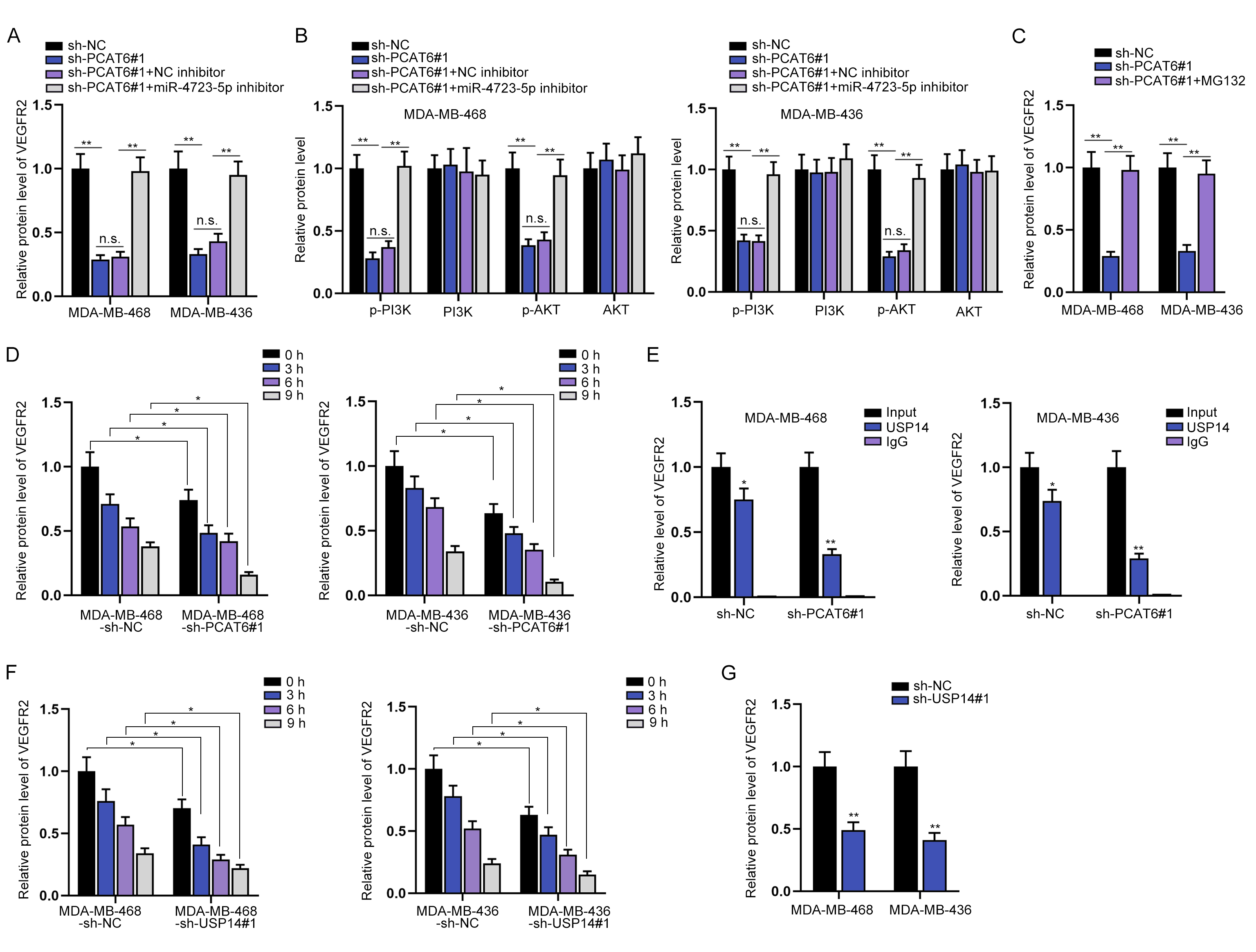

Supplement: Supplementary file 6 — Supplementary file 2 [file 41419_2020_2926_MOESM6_ESM.tif]

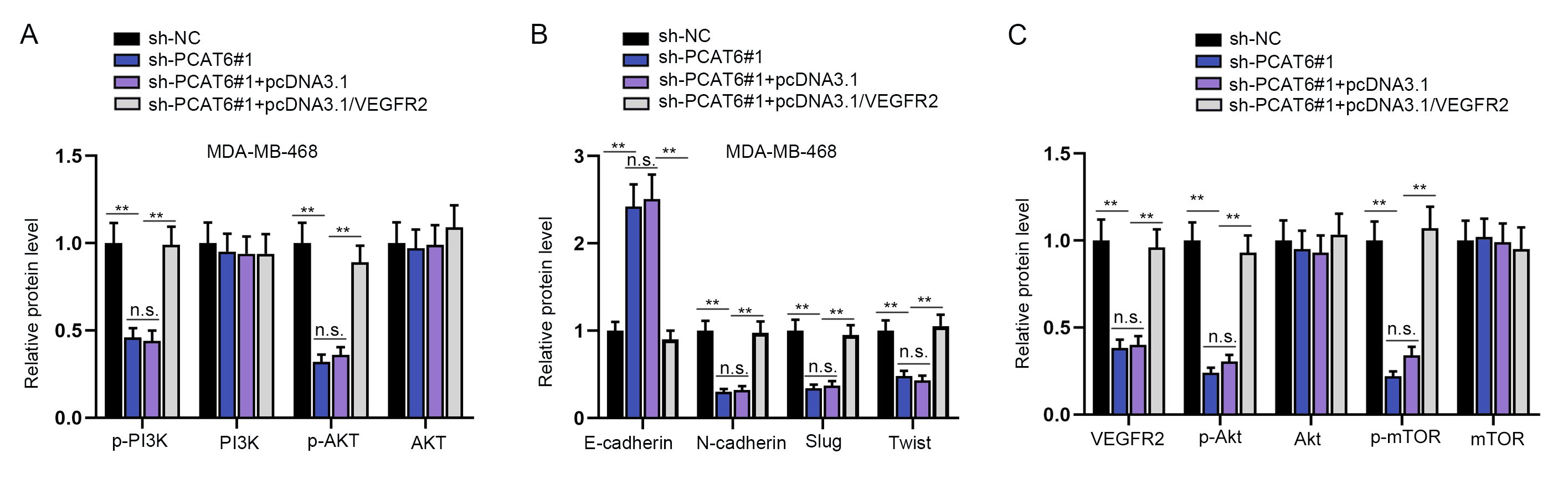

Supplement: Supplementary file 7 — Supplementary file 3 [file 41419_2020_2926_MOESM7_ESM.tif]
