# Supplementary material for: M2 macrophage-induced lncRNA PCAT6 facilitates tumorigenesis and angiogenesis of triple-negative breast cancer through modulation of VEGFR2
Source: Cell Death Dis. 2020 Sep 9;11(9):728. doi: 10.1038/s41419-020-02926-8 (PMC7481779; doi:10.1038/s41419-020-02926-8)
Supplement: Supplementary file 3 — Supplementary figures legends [file 41419_2020_2926_MOESM3_ESM.doc]

**Supplementary figures legends**

**Figure S1. A.** TEM detect the morphology of M2 macrophages-derived exosome. Scale bar = 100 nm. **B.** Expression level of PCAT6 in two TNBC cells treated with or without M2/exosome were detected by qRT-PCR. ^**^P<0.01.

**Figure S2. A.** qRT-PCR verified overexpression efficiency of PCAT6. **B.** 9 miRNAs binding with both VEGFR2 and PCAT6 based on miRmap and lncBase prediction. **C.** qRT-PCR detected the expression of 3 candidate miRNAs in para-carcinoma and TNBC tissues. **D.** Pearson analysis disclosed the correlation between VEGFR2 and PCAT6/ miR-4723-5p in TNBC tissues. **E.** qRT-PCR verified overexpression efficiency of miR-4723-5p. **F.** qRT-PCR detected miR-4723-5p expression in TNBC cells and control cells. **G.** qRT-PCR detected PCAT6 and miR-4723-5p expression. **H.** qRT-PCR verified depletion efficiency of USP14. **I.** qRT-PCR detected influence of USP14 depletion on PCAT6 expression. ^**^P<0.01. n.s.: no significance.
