# Supplementary material for: M2 macrophage-induced lncRNA PCAT6 facilitates tumorigenesis and angiogenesis of triple-negative breast cancer through modulation of VEGFR2
Source: Cell Death Dis. 2020 Sep 9;11(9):728. doi: 10.1038/s41419-020-02926-8 (PMC7481779; doi:10.1038/s41419-020-02926-8)
Supplement: Supplementary file 8 — Supplementary files legends [file 41419_2020_2926_MOESM8_ESM.docx]

**Supplementary files legends**

**Supplementary file 1. A-D.** Quantification graphs for protein bands in Figure 2F, 3B, 3E, 3F. ^**^P<0.01.

**Supplementary file 2. A-G.** Quantification graphs for protein bands in Figure 4G, 4H, 5A, 5B, 5E, 5H, 5I. ^*^P<0.05, ^**^P<0.01. n.s.: no significance.

**Supplementary file 3. A-C.** Quantification graphs for protein bands in Figure 6A, 6F, 7D. ^**^P<0.01. n.s.: no significance.
